# Electrochemical Determination of the “Furanic Index” in Honey

**DOI:** 10.3390/molecules26144115

**Published:** 2021-07-06

**Authors:** Severyn Salis, Nadia Spano, Marco Ciulu, Ignazio Floris, Maria I. Pilo, Gavino Sanna

**Affiliations:** 1 Istituto Zooprofilattico Sperimentale della Sardegna, Via Duca degli Abruzzi 8, 07100 Sassari, Italy; severyn.salis@izs-sardegna.it; 2 Dipartimento di Chimica e Farmacia, Università degli Studi di Sassari, Via Vienna 2, 07100 Sassari, Italy; mpilo@uniss.it (M.I.P.); sanna@uniss.it (G.S.); 3 Department of Animal Sciences, University of Göttingen, Kellnerweg 6, 37077 Göttingen, Germany; marco.ciulu@uni-goettingen.de; 4 Dipartimento di Agraria, Università degli Studi di Sassari, Viale Italia 39/a, 07100 Sassari, Italy; ifloris@uniss.it

**Keywords:** HMF, honey, furanic aldehydes, furanic acids, homogentisic acid, cyclic voltammetry, square wave voltammetry, RP-HPLC

## Abstract

5-(hydroxymethyl)furan-2-carbaldehyde, better known as hydroxymethylfurfural (HMF), is a well-known freshness parameter of honey: although mostly absent in fresh samples, its concentration tends to increase naturally with aging. However, high quantities of HMF are also found in fresh but adulterated samples or honey subjected to thermal or photochemical stresses. In addition, HMF deserves further consideration due to its potential toxic effects on human health. The processes at the origin of HMF formation in honey and in other foods, containing saccharides and proteins—mainly non-enzymatic browning reactions—can also produce other furanic compounds. Among others, 2-furaldehyde (2F) and 2-furoic acid (2FA) are the most abundant in honey, but also their isomers (i.e., 3-furaldehyde, 3F, and 3-furoic acid, 3FA) have been found in it, although in small quantities. A preliminary characterization of HMF, 2F, 2FA, 3F, and 3FA by cyclic voltammetry (CV) led to hypothesizing the possibility of a comprehensive quantitative determination of all these compounds using a simple and accurate square wave voltammetry (SWV) method. Therefore, a new parameter able to provide indications on quality of honey, named “Furanic Index” (FI), was proposed in this contribution, which is based on the simultaneous reduction of all analytes on an Hg electrode to ca. −1.50 V vs. Saturated Calomel Electrode (SCE). The proposed method, validated, and tested on 10 samples of honeys of different botanical origin and age, is fast and accurate, and, in the case of strawberry tree honey (*Arbutus unedo*), it highlighted the contribution to the FI of the homogentisic acid (HA), i.e., the chemical marker of the floral origin of this honey, which was quantitatively reduced in the working conditions. Excellent agreement between the SWV and Reverse-Phase High-Performance Liquid Chromatography (RP-HPLC) data was observed in all samples considered.

## 1. Introduction

**Figure 1:**
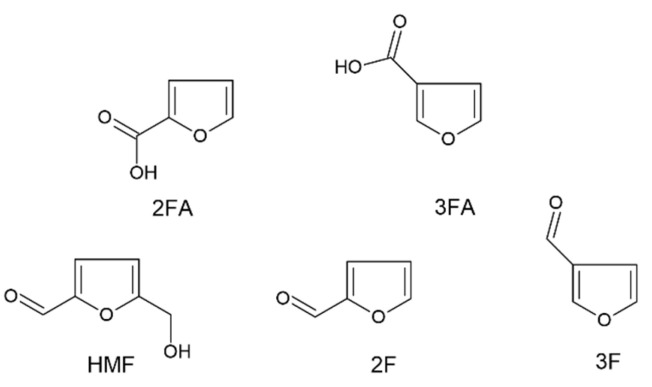
Chemical structures of the furanic acids and aldehydes. 2FA, 2-furoic acid; 3FA, 3-furoic acid; HMF, hydroxymethylfurfural; 2F, 2-furaldehyde; 3F, 3-furaldehyde.

Honey is the sweet natural product that honeybees obtain by conversion of the nectar gathered from flowers. Its composition depends on the geographical, floral, and entomological origins and includes water (15–20%), sugars (80–85% *w*/*w*), nitrogenous compounds, enzymes, phenolic and volatile compounds, organic and amino acids, minerals, and vitamins. On the other hand, it is well known that seasonal, environmental, storage, and processing time and conditions may affect honey’s composition. Although the nutritional and healing features of this food are appreciated, the possible presence of heavy metals (usually in traces) [1], some alkaloids [2], and reactive organic compounds, such as aldehydes, might imply a possible threat for the health of consumers. The 5-(hydroxymethyl)furan-2-carbaldehyde, better known as hydroxymethylfurfural (HMF), is produced during the Maillard reaction and, in honey, acidic conditions and the predominance of monosaccharides, as fructose and glucose, promote its formation during aging or processing [3,4]. For these reasons, the amount of HMF in honey is considered a freshness parameter: while it is basically absent in fresh and well-preserved honey, its amount naturally increases with aging and particularly in the presence of other factors such as heat, sunlight, and metal ions, which promote the course of the Maillard’s reaction [5,6,7]. As a consequence, the Codex Alimentarius Standard commission fixed the maximum content of HMF in honey at 40 mg kg^−1^ (80 mg kg^−1^ for honey produced in tropical regions) and the legislative directives of many countries transposed these indications (Council Directive 2001/110/EC of 20 December 2001 relating to honey). The growing attention of the scientific community toward the effects of HMF helped to reveal not only potential adverse effects on human health, such as cytotoxicity, mutagenicity, chromosomal aberrations, and carcinogenicity [8,9,10], but also positive effects such as antioxidative [11], anti-allergic [12], anti-inflammatory [13], anti-hypoxia [14], anti-sickling [15], and anti-hyperuricemic properties [16]. Hence, the need for accurate methods devoted to the quantitative analysis of HMF in honey is an increasingly relevant target in the field of the quality assurance in hive products and, more in general, for food risk assessment. Three protocols for the determination of HMF in honey are recommended by the International Honey Commission [17]; two of them are spectrophotometric methods (White [18] and Winkler [19]), whereas the last is a reverse-phase high-performance liquid chromatographic (RP-HPLC) method [20]. Both spectrophotometric methods are scarcely sensitive and accurate; in addition, the Winkler method requires the use of the carcinogen *p*-toluidine. On the other hand, the RP-HPLC determination is more accurate than the spectrophotometric ones but it is quite slow. In addition to these, other procedures based on separation techniques, such as RP-HPLC-UV [21] or micellar electrokinetic capillary chromatography (MEKC) [22], electrochemical methods, such as differential pulse polarography (DPP) [23] and square wave voltammetry (SWV) [24,25], or sensors [26,27] were used. Spectroscopic approaches were also reported, including a Winkler-based automated flow injection protocol [28] or a recent method based on the HMF derivatization with the Seliwanoff reagent [29]. Albeit often in minor amounts than HMF, other related species, such as 2-furaldehyde (2F), 3-furaldehyde (3F), 2-furoic acid (2FA), and 3-furoic acid (3FA) may be formed by the degradation processes of honey, hence contributing to the minority composition of reactive organic species present in it. While 2F and 2FA derive, in variable amounts, from the hydrolysis [30] and oxidative degradation of ascorbic acid [31], respectively, the origin of both 3F and 3FA is still debated [32,33]. The literature’s contributions devoted to the contemporary determination of HMF and of the minority furanic aldehydes and acids are rare. All—or some—of these compounds have been measured only using HPLC methods in matrices such as honey [32,33] or in fruit juices and drinks [34]. Despite their simplicity, sensitivity, and accuracy, electrochemical methods have never been used to the best of our knowledge in the comprehensive determination of HMF and of the related furanic aldehydes and acids. Hence, the principal goal of this contribution has been to develop, validate, and apply to real samples a rapid, sensitive, and reliable electrochemical method aimed to contemporaneously determine the total amount of HMF, 2F, 3F, 2FA, and 3FA in honey. Figure 1 reports the chemical structures of all these analytes. To do this, a preliminary evaluation of the electrochemical behavior of all analytes has been accomplished using different techniques (i.e., CV and SWV) in different solvent/supporting electrolyte couples and using different working electrodes (WE). Since the results of the preliminary experimental evidences, an SWV method, aimed to the contemporary determination of HMF, 2F, 3F, 2FA, and 3FA, has been developed, and a new freshness parameter for honey, the so-called “Furanic Index” (FI), may therefore be proposed. The method has been optimized, validated in terms of limit of detection (LoD) and of quantification (LoQ), linearity, precision, and trueness, and tested on a number of real samples of unifloral and blossom honeys, which are different also for age and geographical origin.

## 2. Results and Discussion

### 2.1. Cyclic Voltammetry

#### 2.1.1. Electrochemical Behavior of HMF

The electrochemical behavior of HMF was studied in cyclic voltammetry by varying the nature of the WE (Pt, GC, Au, Hg), the solvent system (solvents: water or methanol; supporting electrolytes: NaH_2_PO_4_, Na_2_HPO_4_, LiCl, CH_3_COONa, NaHCO_3_, LiClO_4_, [(C_4_H_9_)_4_N]ClO_4_) and the potential scan rate (between 0.05 and 2.0 V s^−1^). Based on the cathodic nature of the responses of the analyte, a preliminary evaluation has allowed to discard both Pt and Au working electrodes. Hence, only Hg and GC electrodes were further considered in the optimization of the experimental conditions, as summarized below in Table 1.

**Figure 2:**
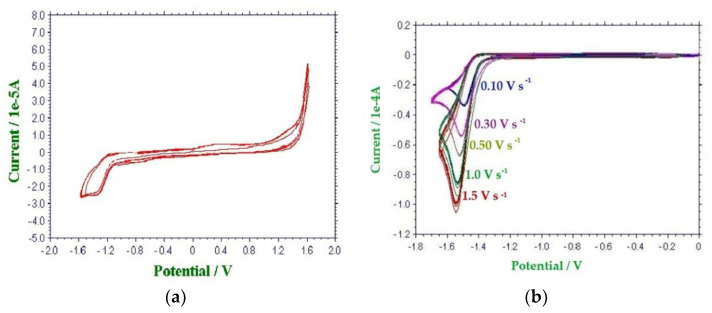
CV responses of HMF (1 mmol dm^−3^) in a 0.1 mol dm^−3^ water solution of supporting electrolyte. AE: Pt, RE: SCE. (**a**) WE: GC, supporting electrolyte: NaH_2_PO_4_, potential scan rate: 0.10 V s^−1^ (**b**) WE: Hg, supporting electrolyte: LiClO_4_, potential scan rate: between 0.10 and 1.5 V s^−1^.

The CV response of an aqueous 1 mmol dm^−3^ solution of HMF showed a well-defined and reproducible irreversible cathodic peak at potentials between −1.10 and −1.55 V vs. SCE, as a function of the nature of both the WE and of the supporting electrolytes (Table 2 and Figure 2).

No anodic peak was observed in anodic direct scan (WE = GC) or after the inversion of the scan direction after crossing the cathodic peak (Figure 2a). Voltammograms acquired using Hg as WE at scan rates between 0.10 and 1.5 V s^−1^ showed a cathodic shift of E_p,c_ (from −1.48 to −1.54 V vs SCE) and a linear increase of the peak’s current (from −3 × 10^−5^ to −1 × 10^−4^ A) (Figure 2b). No backward anodic peak has been observed in this range of scan rates. The linear decreasing of the i_p,c_/√(logV_scan_) ratio as a function of the cathodic shift of the E_p,c_ peak suggests a kinetic control on the overall electrochemical process [35].

**Figure 3:**
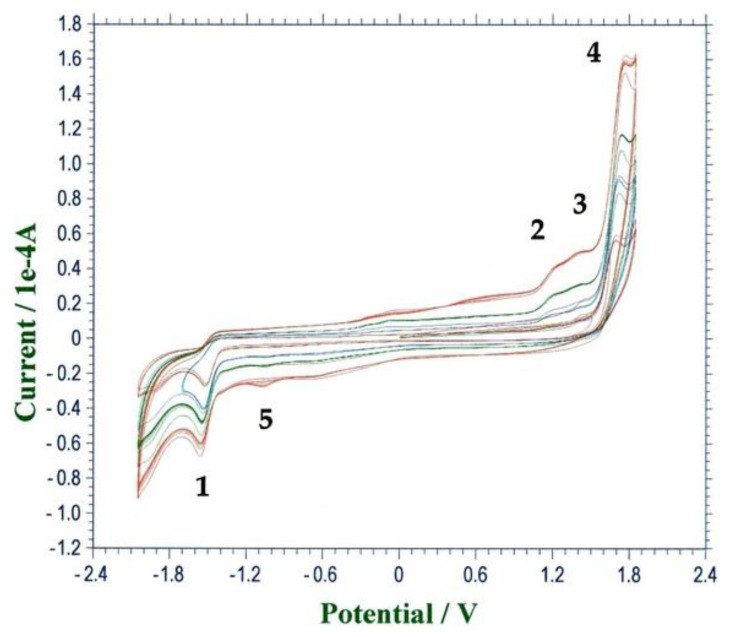
CV responses of HMF (1 mmol dm^−3^) in a 0.1 mol dm^−3^ of LiClO_4_ in methanol. WE: GC; AE: Pt; RE: SCE, potential scan rate: 0.10 V s^−1^, red line; 0.30 V s^−1^, blue line, 0.50 V s^−1^, green line; 1.0 V s^−1^, brown line.

The replacement of water with methanol as solvent did not cause meaningful changes in the morphology of the main cathodic peak previously observed. Indeed, in these conditions, the CV responses recorded on GC, in cathodic direct scan, showed the same irreversible peak (peak **1** in Figure 3) at about −1.50 V vs. SCE. When the scan direction was inverted (from −2.0 to 2.0 V), three ill-resolved and consecutive irreversible anodic peaks became evident (Figure 3, peaks 2, 3 and 4, respectively, at E_p,a_ between 0.90 and 1.70 V vs. SCE). A further inversion of the direction of the potential scan, performed just after peak 4, caused the appearance of a low and irreversible cathodic peak at E_p,c_ of −1.00 V (peak 5 in Figure 3), which was never observed in the cathodic direct scan (Figure 3).

The voltammetric responses of the solutions of HMF were in agreement with those reported by other authors who studied this analyte’s behavior in aqueous solutions [25] as well as the reduction processes that involve the carbonylic group in 2F [35,36]. All the authors attributed the irreversible cathodic peak at about −1.50 V to the two-electron reduction of the aldehydic group to the corresponding alcohol, that, in the case of HMF, produces the 2,5-dihydroxymethylfurane. In addition, the meaningful cathodic shift of this irreversible reduction peak, observed passing by a solvent system at pH = 4.1 (supporting electrolyte: NaH_2_PO_4_) to that at pH = 9.8 (supporting electrolyte: Na_2_HPO_4_), is consistent with the observations of Ganesan et al. [36], because low pH values assist the reduction process. On the other hand, the most anodic peak, observed at 1.68 V vs. SCE in the direct anodic scan on GC in the methanol/LiClO_4_ solvent system, was previously attributed to the irreversible oxidation of HMF to 2,5-diformylfurane (1.63 V vs. SCE, [25]), which was likely reducible, in the reverse cathodic scan, to HMF (cathodic peak at −1.00 V vs. SCE). Since the reduction of carbonylic group of HMF is the main (often the unique) peak and the most reproducible one present in the different solutions, we decided to further investigate it to find the optimal conditions for its quantitative determination in honey samples.

#### 2.1.2. Electrochemical Behavior of Furanic Aldehydes and Acids

The electrochemical behavior of 2F, 3F, 2FA, and 3FA was studied in CV in the same conditions used for HMF. Table 3 reports the potentials of the cathodic peak measured using Hg and GC as working electrodes, and LiCl, LiClO_4_, CH_3_COONa, and Na_2_HPO_4_ as supporting electrolytes in water. All the species showed an irreversible cathodic reduction peak at potential between −1.46 and −1.84 V vs. SCE, using both the working electrodes, when LiCl and LiClO_4_ were used as supporting electrolytes. These peaks can be tentatively ascribed to the reductions of 2F and 3F to give the corresponding alcohols (i.e., 2- and 3-hydroxymethylfurane, respectively) and the reductions of 2FA and 3FA toward 2F and 3F, respectively. On the other hand, the cathodic shift of the reduction process caused by the use of alkaline supporting electrolytes brought the E_p,c_ of 2FA and 3FA out of the available cathodic window. This happened when the GC working electrode was used in the presence of CH_3_COONa as supporting electrolyte and, for both Hg and GC electrodes, when Na_2_HPO_4_ was used. Hence, the possibility to measure at the same time all these analytes in honey is achievable only using neutral supporting electrolytes such as LiCl or LiClO_4_.

### 2.2. Square Wave Voltammetry

#### Method Optimization

Preliminary CV experiments allowed to ascertain the feasibility in a contemporary determination of HMF and of the principal furanic aldehydes and acids in honey. Unfortunately, CV is an unsuitable electrochemical technique for an accurate and sensitive determination of trace compounds. On the other hand, the amount of the most abundant of these analytes in fresh honey, the HMF, is typically of few tens of mg kg^−1^, i.e., well below the concentration used for the preliminary CV tests. Hence, the quantitative method to be assessed had to be able to reach LoQ levels at least of few mg kg^−1^, and the best choice to quantify trace amounts of electroactive organic analytes was the square wave voltammetry (SWV). The method has been optimized as a function of the general parameters, such as the nature of the working electrode (Hg or GC) and of the supporting electrolyte (LiCl or LiClO_4_), as well as of specific SWV parameters, such as the pulse height (values between 2.5 and 250 mV), the frequency (between 1.5 and 150 Hz), and the modulation amplitude (between 1 and 40 mV). The optimized SWV parameters are pulse height, 4 mV; frequency, 15 Hz; modulation amplitude, 25 mV. Table 4 shows the optimization of both WE and supporting electrolyte in the development of the SWV method. HMF was chosen as a molecular benchmark, since it is the most abundant furanic compound present in honey.

Despite the higher sensitivity of the GC electrode, when LiClO_4_ was the supporting electrolyte, no response was obtained using LiCl. On the other hand, the Hg electrode could detect the benchmark HMF content using both the supporting electrolytes. In these cases, the density of current measured in the water/LiCl solvent system was roughly double the amount measured in water/LiClO_4_. Based on the results here obtained, the SWV method was tested also in the presence of 2F, 3F, 2FA, and 3FA. Additionally in this case, two WEs and two supporting electrolytes were used in these experiments. Table 5 reports the electrochemical parameters obtained for all the analytes in this cycle of measurements.

**Figure 4:**
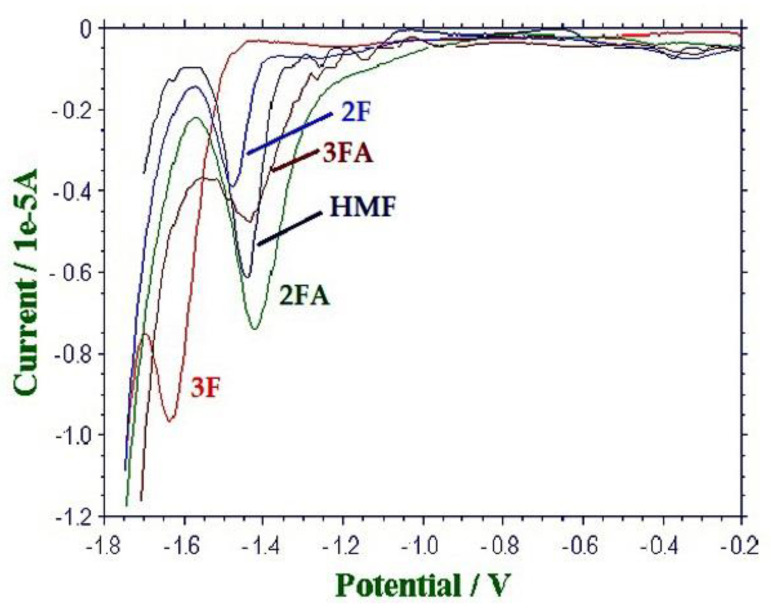
SW voltammograms of: HMF (0.08 mmol dm^−3^), 2F (0.10 mmol dm^−3^), 3F (0.62 mmol dm^−3^), 2FA (0.36 mmol dm^−3^), and 3FA (0.18 mmol dm^−3^) in 0.1 mol dm^−3^ LiCl aqueous solution, WE: Hg; AE: Pt; RE: SCE. Potential scan rate: 0.10 V s^−1^; pulse height: 4 mV; frequency: 15 Hz; modulation amplitude: 25 mV.

Data reported in Table 5 confirmed the preliminary results obtained for HMF. The E_p,c_ values for the five analytes measured with Hg electrode were more reproducible and closer to each other in LiCl rather than in LiClO_4_. In addition, it was impossible to detect the cathodic peak of 3FA with GC electrode, when LiClO_4_ was used as supporting electrolyte. Furthermore, the current densities measured for all analytes, when Hg was the working electrode, were much higher than those measured by GC electrode, and the same happened switching from LiClO_4_ to LiCl as the supporting electrolyte. Hence, Hg as the working electrode and LiCl as the supporting electrolyte were the best choices for the SWV determination of HMF, 2F, 3F, 2FA, and 3FA in honey. If the extreme closeness of all the E_p,c_ values made it almost impossible to discriminate the amount of each analyte, individually, as clearly demonstrated by Figure 4, it was however possible—and easy—to quantify the amounts of all these analytes at once, expressing them in terms of an equivalent amount of HMF. This observation allowed defining the “Furanic Index” (FI) as a freshness-related parameter for honey that is able to evaluate the overall amount of HMF, 2F, 3F, 2FA, and 3FA, as expressed in mg of HMF per kg of honey. From a quantitative viewpoint, the expression of FI is:

FI (mg dm^−3^) = C_HMF_ + C_2F_·(HMF/2F) + C_3F_·(HMF/3F) + C_2FA_·(HMF/2FA) + C_3FA_·(HMF/3FA)

where C_analyte_ is the amount (in mg dm^−3^) of each furanic compound, and the HMF/analyte ratio refers to the relevant molecular weights.

### 2.3. Validation

Validation of the proposed method has been accomplished in terms of LoD, LoQ, linearity, precision, and trueness. Table 6 reports the features describing the performances of the method proposed.

#### 2.3.1. LoD and LoQ

These parameters were calculated, according to IUPAC recommendation, through the ULA1 method [37]. Four different 0.1 mol dm^−3^ LiCl aqueous solutions containing HMF in concentrations of 3 mg dm^−3^, 5 mg dm^−3^, 7 mg dm^−3^, and 10 mg dm^−3^, respectively, were prepared and analyzed by means of SWV in triplicate. The LoD obtained in this way is 0.6 mg dm^−3^. This amount is significantly higher than that (i.e., 0.0021 mg dm^−3^) recently measured by Sahli et al. [25], but in this case, the approach used for the calculation was not described. On the other hand, it is well known that in the past, it has been possible to associate to the general expression “detection limit” values spanning over more than three orders of magnitude [38]. Hence, even without arguing on the overall reliability of very low LoDs reported in the literature, it is well known that the application of the ULA methods, albeit providing sometimes LoDs higher than those obtained by more “optimistic” approaches [33], is one of the best choices to avoid both type-1 and type-2 decision errors. For the ULA1 approach, LoQ is three times the LoD value, i.e., 1.8 mg dm^−3^.

#### 2.3.2. Linearity

Linearity was measured over a concentration range between LoQ and 200 mg dm^−3^. This is the typical interval of amounts observed for HMF in honeys of different geographical origin and age. As shown in Table 6, very good correlation coefficients (R^2^) were observed in this case. In addition, no “hidden” deviations from linearity have been evidenced by the output of the graphical analysis performed on the residuals of the regression line.

#### 2.3.3. Precision

This parameter was evaluated on real samples in terms of repeatability and intermediate precision, which are both expressed as the percent relative standard deviation (RSD%). Repeatability was obtained by analyzing five times, in the same analytical session, four honey samples with a concentration range between 4.8 and 306 mg kg^−1^. On the other hand, intermediate precision was evaluated by analyzing five times, in five different analytical sessions over five months, a honey sample showing an intermediate value of FI. As a function of the FI in the honey samples, the RSD% of repeatability ranged between 0.72% (FI of 306 mg kg^−1^) and 9.9% (FI of 4.8 mg kg^−1^), whereas the RSD% of the intermediate precision was of 3.0%, which was measured on a honey showing an FI of 160 mg kg^−1^. All precision parameters were acceptable according to the Horwitz’s theory [39].

#### 2.3.4. Trueness

Given the absence of any Certified Reference Materials, but in consideration that an independent analytical method is currently available [33], trueness was evaluated through the comparison of results obtained from five real samples by both the proposed method and the RP-HPLC method previously published by this research group, which is able to singularly quantify HMF, 2F, 3F, 2FA, and 3FA in the same chromatographic run. The comparison of the experimental t (between 0.55 and 2.19) with the tabulated t (2.57 for 5 degrees of freedom, *p* = 0.95 in a two-tail *t*-test) allowed concluding that the proposed method was bias-free.

### 2.4. SWV Determination of the Furanic Index in Honeys

#### 2.4.1. Method Application

**Figure 5:**
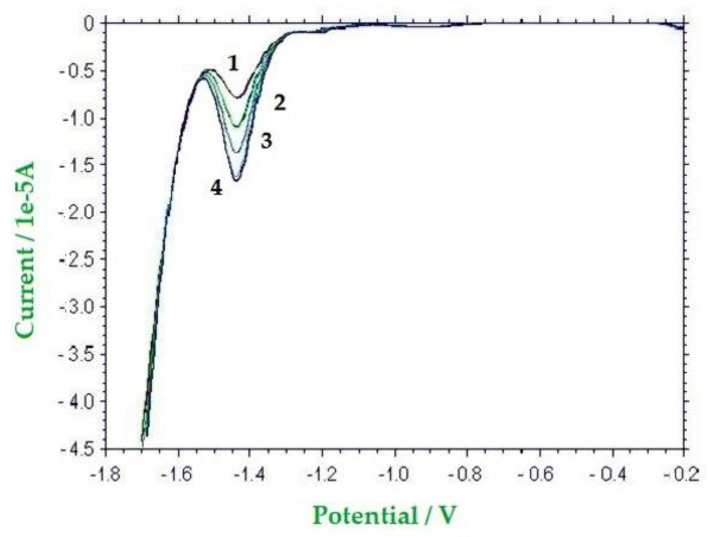
SW voltammograms of (1) a pure strawberry tree honey (50% *w*/*w*) in a 0.1 mol dm^−3^ LiCl solution in water; (2) the same solution 1, after the addition of 0.75 mg of HMF, (3) the same solution **1**, after the addition of 1.50 mg of HMF; (4) the same solution 1, after the addition of 2.25 mg of HMF. WE: Hg; AE: Pt; RE: SCE. Potential scan rate: 0.10 V s^−1^; pulse height: 4 mV; frequency: 15 Hz; modulation amplitude: 25 mV.

The proposed method was tested on ten honey samples of different botanical origin (three from blossom, one from eucalyptus, one from thistle, and five from strawberry tree) and of different age (between 2014 and 2019). Figure 5 reports a typical SW voltammogram for one real sample.

#### 2.4.2. Furanic Index in Honeys from Different Botanical Origin and Age: Comparison with HPLC Data

Table 7 reports the amounts of FI measured in these samples. For comparison purposes, the last column of Table 7 also reported the sum of all analytes (i.e., HMF, 2F, 3F, 2FA, and 3FA) measured in the same samples by means of a literature RP-HPLC method [33], which are all expressed in equivalent amounts of HMF. It is possible to observe that the FI measured on blossom, eucalyptus, and thistle honey is never statistically different (two tails *t*-test, *p* = 0.95) from the amount measured by the RP-HPLC method. This fact is relevant, as it shows that the quicker SWV determination of the total amount of the furanic compounds is effective in the assessment of the freshness of the honey samples. In particular, RP-HPLC data substantiated the expected large predominance of the amount of HMF on those of the other analytes. As a matter of fact, HMF constitutes on average 93% of the total furanic species in these honeys. 2F and 2FA were sometimes quantified, at concentrations never higher than 3.0 mg kg^−1^ and 4.0 mg kg^−1^, respectively, while 3F and 3FA were always below the relevant LoQs (i.e., 0.3 mg kg^−1^ and 0.09 mg kg^−1^, respectively). The furanic indexes measured in these samples were always congruent to a recently produced and well-preserved honey: indeed, the measured FI ranged between 4.8 ± 0.4 mg kg^−1^ (B3 honey) and 22.0 ± 0.5 mg kg^−1^ (E honey).

**Figure 6:**
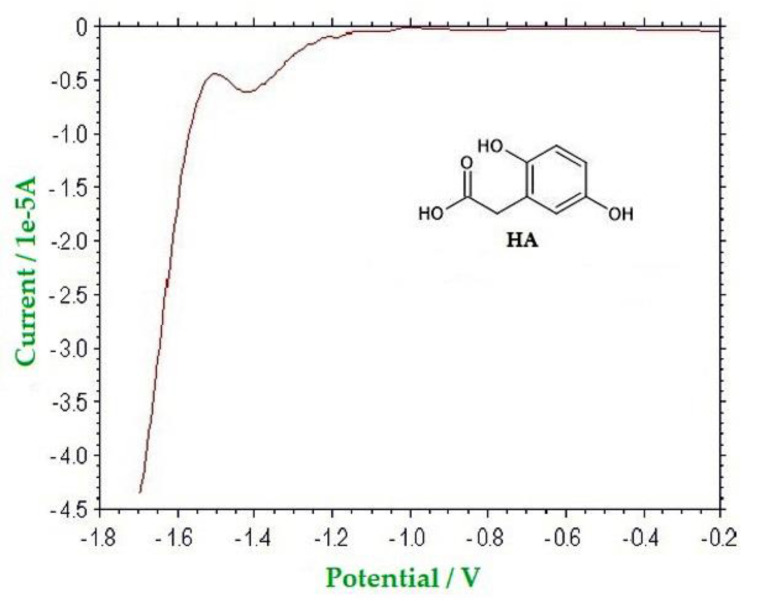
SW voltammogram of HA (0.1 mmol dm^−3^) in 0.1 mol dm^−3^ LiCl aqueous solution, WE: Hg; AE: Pt; RE: SCE. Potential scan rate: 0.10 V s^−1^; pulse height: 4 mV; frequency: 15 Hz; modulation amplitude: 25 mV.

On the other hand, a very different situation was exhibited by the five strawberry tree honeys: in comparison to the RP-HPLC data, the amount of the FI is much higher (from 191% to 350%) than the sum of furanic species measured by RP-HPLC. The fact that this result only occurred for the samples of this botanical origin—irrespectively of the age of the honey led to supposing that in this honey, a compound normally absent in the other honeys was present, which was reduced at the same potential of all the analytes considered. As a matter of fact, the strawberry tree honey is rich in 2,5-dihydroxyphenylacetic acid, homogentisic acid (HA), and L-tyrosine catabolite [40], and this compound has been found until now only in this honey [41,42]. The electrochemical behavior of HA was studied in the same conditions used for the characterization of all the furanic compounds, and the voltammetric evidences acquired both in CV and in SWV confirmed that it is irreversibly reduced in the cathodic direct scan, at −1.48 V vs. SCE. Figure 6 shows a SW voltammogram of a solution 0.1 mol dm^−3^ of HA.

To verify the contribution of this molecule to the FI measured in strawberry tree honeys, HA was quantified in all the samples of this botanical origin by means of another RP-HPLC literature method [42]. The amounts of HA ranged between 92.5 ± 0.2 mg kg^−1^ and 241.2 ± 0.5 mg kg^−1^, as shown in Table 8. This table also reports the theoretical amount of reducible species present in the strawberry tree honey, as measured by RP-HPLC, and expressed in equivalent HMF concentration, column RP-HPLC ΣHMF+Fs+FAs+HA. It is interesting to note that—for honey samples S1 and S3—this amount is not statistically different from that measured by SWV (criteria: two tails t-test, *p* = 0.95), whereas for the remaining samples, these data exhibit a slight bias (between −3.16%, sample S5 and −6.43%, sample S4) in comparison to the RP-HPLC one. However, this bias is well within the acceptability range for this level of concentration, as suggested by the AOAC International in its Peer Verified Methods Programs [43].

## 3. Materials and Methods

### 3.1. Honey Samples

The study was carried out on 10 honey samples of different botanical origin: strawberry tree (*Arbutus unedo*), eucalyptus (*Eucalyptus camaldulensis*), thistle (*Galactites tomentosus*), and blossom. Some of these honeys were directly provided by beekeepers; the others were commercial samples. All the samples were produced in Italy, almost all in the Sardinia Island, between 2014 and 2019. The floral origin was established on the basis of the indications of the producers and verified by melissopalynological analysis [44]. All samples were stored in the dark at 4 °C until SWV analysis.

### 3.2. SWV Determination of FI in Honey Samples

Ca. 5 g (exactly weighted on an analytical balance) of homogenized honey was diluted to 10 cm^3^ by a 0.1 mol dm^−3^ LiCl aqueous solution and transferred in a polarographic cell in glass equipped with a four-hole gas-tight cover. The solution was carefully deaerated with Ar for at least 15 min; hence, a gentle flow of Ar ensured an inert blanket over the solution during the tests. Quantification was performed by internal calibration, by means of the multiple additions method (i.e., with three consecutive spikings of HMF, respectively 50%, 100%, and 150% of the amount of the furanic index evaluated by measurements performed by a literature HPLC method [33]). Each analytical evaluation was replicated at least three times.

### 3.3. Chemicals and Reagents

2-furoic acid, >98%; 3-furoic acid, ≥98%; 2-furaldehyde, >99%; 3-furaldehyde, ≥97%; 5-hydroxymethylfurfural, ≥99%; 2,5-dihydroxyphenylacetic acid, ≥99%; mercury, 99.9995%; methanol anhydrous, >99.8%; methanol HPLC grade; 1.0000 mol dm^−3^ H_2_SO_4_ solution in water and type I water were purchased from Merck, Milan, Italy. In addition, lithium perchlorate, 99.99%; lithium chloride, 99.98%; sodium hydrogen carbonate, >99.7%; sodium acetate, >99.9%; disodium hydrogen phosphate, 99.99%; sodium dihydrogen phosphate, 99.99%; tetrabutylammonium perchlorate, >99%, all used as supporting electrolytes were analytical grade Merck reagents. 99.999% Ar gas was used for degassing the solvent systems used in all electrochemical experiments and was purchased from Sapio, Monza, Italy.

### 3.4. Equipment

All the electrochemical measurements were performed using a CHI-650 electrochemical workstation (CH Instruments, Austin, TX, USA) interfaced with a computer with the specific software CHI-650, in a conventional three-electrodes voltammetric cell in glass, equipped with a gas-tight four-hole cover, positioned inside a Faraday cage. Four different working electrodes (WE) were used in this study: a 2 mm diameter glassy carbon (GC) disk, a 2 mm diameter Pt disk, a ca. 1 mm diameter Au hemisphere electrode, and a ca. 1 mm diameter Hg/Au amalgam hemisphere electrode, respectively. A 5 mm Pt disk was always the auxiliary electrode (AE), and a saturated calomel electrode (SCE) was the reference electrode (RE); all potential values given were referred. Before use, all of the electrode surfaces of WE and AE were polished with alumina powder (0.3 μm of diameter) and rinsed with ultrapure water. All the experiments were performed at room temperature in an Ar saturated solution.

The HPLC equipment used for confirmatory purposes was a Series 200 apparatus (Perkin Elmer, Milan, Italy) formed by a binary pump, by an UV–vis variable wavelength detector, and by a sampling valve equipped with a 20 mm^3^ sample loop. Separation was accomplished on an Alltima C18 column 250 mm × 4.6 mm, 5 μm particle size (Alltech, Sedriano, Italy) fitted with a guard cartridge packed with the same stationary phase. Data were processed by a Turbochrom Workstation Software (Perkin Elmer, Milan, Italy).

An Ultra-turrax mixer model T18 (IKA, Staufen, Germany) was used to homogenize honey samples before analysis.

## 4. Conclusions

Although HMF has been a well-recognized freshness index of honeys for years, it is possible that the aging and/or imperfect storage conditions allow the formation also of different furanic compounds, such as furaldehydes and furoic acids. In order to provide a thorough evaluation of any reactive furanic-based compounds formed in honeys by degradation of this matrix, a rapid and accurate SWV method has been developed, optimized, and validated to measure the amount of HMF as well as of 2-furaldehyde, 2F, 3-furaldehyde, 3F, 2-furoic acid, 2FA, and 3-furoic acid, 3FA. All these analytes underwent an irreversible reduction on Hg electrode at potentials close to −1.50 V vs. SCE. Hence, a new chemical index (i.e., the so-called “Furanic Index”, FI) was proposed for a quick and accurate assessment of the freshness of honeys. FI expresses, as mg of HMF per kg, the total amount of HMF, 2F, 3F, 2FA, and 3FA found in honey. The method exhibits satisfactory LoD and LoQ values, excellent linearity inside the operative range of concentration (i.e., between 2 and 200 mg dm^−3^). The method was tested on ten real honey samples that were different in age and floral origin. An excellent accuracy has been demonstrated for blossom as well as eucalyptus and thistle honeys, while for strawberry tree samples, homogentisic acid, the chemical marker of this floral origin, also contributed to FI as it was quantitatively reduced in the analytical conditions. However, RP-HPLC determination of HA allows evaluating the freshness of these samples by FI. So, it is possible to believe that the proposed method could be advantageously used for screening purposes due its simplicity and its speed.

## Figures and Tables

**Table 1:** CV operative conditions.

	Operative Parameters
WE	GC disk (ø 2 mm) or Hg hemisphere (ø 1 mm)
RE	SCE
AE Potential scan rate	Pt 0.10 V s^−1^
Supporting electrolytes (0.1 mol dm^−3^ in water)	NaH_2_PO_4_, Na_2_HPO_4_, LiCl, LiClO_4_, CH_3_COONa, NaHCO_3_
Supporting electrolytes (0.1 mol dm^−3^ in methanol)	LiClO_4_, [(C_4_H_9_)_4_N)]ClO_4_
HMF solution	10 cm^3^, 1 mmol dm^−3^

**Table 2:** Cathodic peak potential (E_p,c_) and current density at the cathodic peak (J_p,c_) of the irreversible reduction process of HMF by varying the nature of the WE (Hg or GC) and the nature and the pH of the solvent system (a 0.1 mol dm^−3^ water solution of five different supporting electrolytes). HMF concentration: 1 mmol dm^−3^, AE: Pt, RE: SCE, potential scan rate: 0.10 V s^−1^.

WE	Hg		GC	
Supporting Electrolytes (pH)	E_p,c_ (V)	J_p,c_ (A mm^−2^)	E_p,c_ (V)	J_p,c_ (A mm^−2^)
NaH_2_PO_4_ (4.1)	−1.10	−3.21 × 10^−3^	−1.35	−7.93 × 10^−4^
LiCl (7.0)	−1.48	−1.02 × 10^−3^	−1.52	−2.58 × 10^−5^
LiClO_4_ (6.9)	−1.48	−3.54 × 10^−5^	−1.52	−3.18 × 10^−5^
CH_3_COONa (8.8)	−1.46	−4.59 × 10^−5^	−1.52	−8.40 × 10^−6^
Na_2_HPO_4_ (9.8)	−1.48	−3.09 × 10^−5^	−1.55	−8.82 × 10^−6^

**Table 3:** Cathodic peak potentials E_p,c_ (V vs. SCE) for HMF, 2F, 3F, 2FA, and 3FA measured in a CV direct cathodic scan on a 0.1 mol dm^−3^ aqueous solution of four different supporting electrolytes. Analyte concentration: 1 mmol dm^−3^, potential scan rate: 0.10 V s^−1^, AE: Pt, RE: SCE.

	Supporting Electrolyte								
	LiCl			LiClO_4_		CH_3_COONa		Na_2_HPO_4_	
	WE (E_p,c_, V)			WE (E_p,c_, V)		WE (E_p,c_, V)		WE (E_p,c_, V)	
Analyte	Hg	GC	Hg		GC	Hg	GC	Hg	GC
HMF	−1.48	−1.54	−1.48		−1.52	−1.46	−1.52	−1.48	−1.55
2F	−1.49	−1.54	−1.50		−1.55	−1.48	−1.54	−1.47	−1.49
3F	−1.68	−1.76	−1.65		−1.76	−1.67	−1.74	−1.64	−1.69
2FA	−1.46	−1.78	−1.48		−1.84	−1.64	nd	nd	nd
3FA	−1.48	−1.76	−1.49		−1.77	−1.61	nd	nd	nd

nd: no reduction process has been observed within the cathodic window available for the WE/solvent system couple.

**Table 4:** Cathodic peak potentials E_p,c_ (V vs. SCE) and cathodic peak current densities J_p,c_ (μA mm^−2^) in the SWV determination of HMF (concentration: 16 μmol dm^−3^) by varying the nature of WE (Hg or GC) and that of the supporting electrolyte (LiCl or LiClO_4_, both 0.1 mol dm^−3^ in water). Potential scan rate: 0.10 V s^−1^; cathodic window of potential: between 0 and −2.00 V vs. SCE; AE: Pt; RE: SCE; pulse height: 4 mV; frequency: 15 Hz; modulation amplitude: 25 mV.

WE	Hg		GC	
Supporting Electrolyte	E_p,c_ (V)	J_p,c_ (μA mm^−2^)	E_p,c_ (V)	J_p,c_ (μA mm^−2^)
LiCl	−1.43	−0.152	nd	nd
LiClO_4_	−1.45	−0.072	−1.53	−0.164

**Table 5:** Cathodic peak potentials E_p,c_ (V vs. SCE) and cathodic peak current densities J_p,c_ (μA mm^−2^) in the SWV determination of HMF, 2F, 3F, 2FA, and 3FA (concentration: 1 mmol dm^−3^ each) by varying the nature of WE (Hg or GC) and that of the supporting electrolyte (LiCl or LiClO_4_, both 0.1 mol dm^−3^ in water). Potential scan rate: 0.10 V s^−1^; cathodic window of potential: between 0 and −2.0 V vs. SCE; AE: Pt; RE: SCE; pulse height: 4 mV; frequency: 15 Hz; modulation amplitude: 25 mV.

	Supporting Electrolyte							
	LiCl				LiClO_4_			
	WE = Hg		WE = GC		WE = Hg		WE = GC	
Analyte	E_p,c_ (V vs. SCE)	J_p,c_ (μA mm^−2^)	E_p,c_ (V vs. SCE)	J_p,c_ (μA mm^−2^)	E_p,c_ (V vs. SCE)	J_p,c_ (μA mm^−2^)	E_p,c_ (V vs. SCE)	J_p,c_ (μA mm^−2^)
HMF	−1.43	−77.1	−1.47	−13.7	−1.45	−54.9	−1.48	−10.4
2F	−1.47	−33.4	−1.49	−4.55	−1.47	−2.98	−1.51	−0.48
3F	−1.61	−13.1	−1.70	−1.16	−1.62	−2.53	−1.70	−3.34
2FA	−1.42	−21.3	−1.70	−1.46	−1.43	−1.69	−1.84	−0.32
3FA	−1.44	−14.0	−1.70	−1.96	−1.44	−9.57	nd	nd

nd: no reduction process has been observed within the cathodic window available for the WE/solvent system couple.

**Table 6:** Validation parameters for the SWV determination of the furanic index (FI) in honey.

LoD	LoQ	Linearity	Repeatability ^3^	Intermediate Precision ^4^	Trueness ^5^
		Concentration range: 2–200 mg dm^−3^	RSD% (FI, mg kg^−1^)	RSD% (FI, mg kg^−1^)	Two tails *t*-test (*p* = 0.95)
			9.9 (4.8)		
0.6 mg dm^−3, 1^	1.8 mg dm^−3, 1^	Y = (a ± s_a_)X + (b ± s_b_)			t_tab_ = 2.57
		a = 1.07 × 10^−7^	4.4 (22)		
		s_a_ = 0.01 × 10^−7^		3.0 (160)	t_exp_ < 2.19
1.2 mg kg^−1, 2^	3.6 mg kg^−1, 2^	b = 2 × 10^−7^	2.3 (120)		
		s_b_ = 1 × 10^−7^			
		R^2^ = 0.9991	0.72 (306)		

The LoD value was calculated according to [37]. ^1^ LoD calculated on a 50% solution of honey in a 0.1 mol dm^−3^ LiCl/water solvent system; ^2^ LoD calculated on pure honey; ^3^ parameter evaluated by analyzing four different honey samples five times in the same analytical session; ^4^ parameter evaluated by analyzing five times in five different analytical sessions over five months a honey sample exhibiting an intermediate FI with respect the concentration range measured; ^5^ evaluated by means of comparison with an independent analytical method (i.e., the RP-HPLC method, [33]) made on five different honey samples.

**Table 7:** Furanic index (FI) in ten honeys of different floral origin and age. Comparison with RP-HPLC data.

Sample	Year of Production	Floral Origin	SWV FI (mg kg^−1^)	RP-HPLC ^1^ ΣHMF + Fs + FAs (mg kg^−1^)
B1	2018	Blossom	14.2 ± 0.6	13.3 ± 0.2
B2	2019	Blossom	12.0 ± 0.5	10.4 ± 0.2
B3	2019	Blossom	4.8 ± 0.4	3.7 ± 0.1
T	2018	Thistle	19.4 ± 0.8	17.4 ± 0.4
E	2019	Eucalyptus	22.0 ± 0.5	19.5 ± 0.3
S1	2015	Strawberry tree	192 ± 1	79 ± 1
S2	2016	Strawberry tree	245 ± 2	70.4 ± 0.8
S3	2018	Strawberry tree	120 ± 2	52 ± 1
S4	2016	Strawberry tree	160 ± 2	67 ± 2
S5	2014	Strawberry tree	306 ± 2	160 ± 2

All data are expressed as average ± standard deviation, *n* = 3. ^1^ Sum of the concentrations of HMF, of 2F, of 3F, of 2FA and of 3FA, expressed as mg of HMF on kg of honey.

**Table 8:** RP-HPLC determination of homogentisic acid (HA) in the strawberry tree honeys and comparison among results obtained by means of both RP-HPLC and SWV methods.

Sample	RP-HPLC ^1^ HA (mg kg^−^^1^)	RP-HPLC ^2^ ΣHMF + Fs + FAs (mg kg^−1^)	RP-HPLC ^3^ ΣHMF + Fs + Fas + HA (mg kg^−1^)	SWV ΣHMF + Fs + Fas + HA (mg kg^−1^)
S1	150.0 ± 0.1	79 ± 1	194 ± 1	192 ± 1
S2	241.2 ± 0.5	70.4 ± 0.8	256 ± 1	245 ± 2
S3	92.5 ± 0.2	52 ± 1	123 ± 1	120 ± 2
S4	135.3 ± 0.3	67 ± 2	171 ± 2	160 ± 2
S5	202.8 ± 0.2	160 ± 2	316 ± 2	306 ± 2

All data are expressed as average ± standard deviation, *n* = 3. ^1^ Data obtained according [42]; ^2^ sum of the concentrations of HMF, 2F, 3F, 2FA, and 3FA, obtained according to [33] and expressed as mg of HMF on kg of honey; ^3^ sum of the concentrations of HMF, 2F, 3F, 2FA, 3FA, and HA, obtained according to [33,42] and expressed as mg of HMF on kg of honey.

## Data Availability

The data used to support the findings of this study are available from the corresponding author upon request.
